# Comment on: Is “pre-sepsis” the new sepsis? A narrative review

**DOI:** 10.1371/journal.ppat.1013887

**Published:** 2026-03-06

**Authors:** Andres Giglio, Maria Aranda, Eric Macias, Marcio Borges

**Affiliations:** 1 Multidisciplinary Sepsis Unit, Son Llatzer Hospital, Palma, Spain; 2 Multidisciplinary Sepsis Group, Health Research Institute of the Balearic Islands, Palma, Spain; 3 Balearic Islands University, Palma, Spain; 4 Faculty of Medicine, Finis Terrae University, Santiago, Chile; 5 Clinica Las Condes Hospital, Santiago, Chile; 6 Instituto de Ingenieria del Conocimiento, Madrid, España; 7 Fundación Codigo Sepsis, Valencia, Spain; INSERM, FRANCE

## Dear editor

We read with great interest the narrative review by Gerard et al. on the concept of “pre-sepsis” and wish to share our clinical experience that validates this theoretical framework [1].

Since 2019, we have implemented BiAlert Sepsis AI for sepsis prediction in our clinical practice [2]. Analysis of our last 12 months’ data reveals a striking distribution: only a fraction of AI-detected patients fulfill our local sepsis criteria (based on a fusion of modified Sepsis-2 with dysfunction or Sepsis-3 [3,4]) at the moment of detection. While a small proportion are false detections (conditions mimicking sepsis such as acute heart failure or decompensated cirrhosis), critically, most of “non-septic patients” represent infected ones without early organ dysfunction - precisely the “pre-sepsis” population Gerard et al. propose [1].

**Fig 1:**
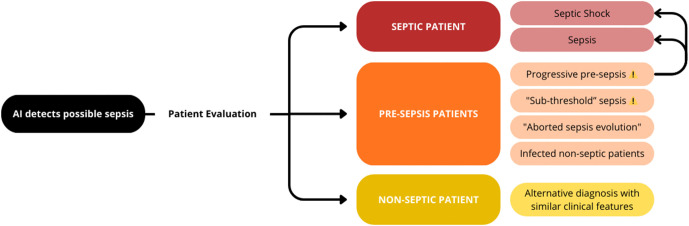
BiAlert Sepsis AI Classification Algorithm identifying pre-sepsis heterogeneity. Flowchart shows four trajectories within the pre-sepsis population. Progressive pre-sepsis (highlighted, with loop arrows) shows patients developing threshold-crossing organ dysfunction within 24-48 hours. “Sub-threshold” patients exhibit genuine systemic alterations constituting pathophysiological dysfunction that fails to meet diagnostic cutoffs. These groups raise whether “pre-sepsis” represents unrecognized early sepsis. Remaining trajectories include aborted sepsis evolution and infected non-septic patients (true “at-risk-of-sepsis” states). Alternative diagnoses represent false-positive detections.

Within this pre-sepsis cohort, we have identified four distinct clinical trajectories (Fig 1):

- **Infected patients at risk who do not develop sepsis**: Predominantly young, immunocompetent individuals meeting SIRS criteria without progressing to organ dysfunction - representing successful immune containment within the pre-sepsis phase.
- **“Aborted sepsis evolution”**: Patients detected before sepsis develops, such as bacteremic patients identified in the critical window before organ dysfunction manifests. This exemplifies the ideal therapeutic opportunity Gerard et al. advocate.
- **“Sub-threshold sepsis patients”**: Those with subtle organ dysfunction signals (SOFA = 1, or values just below diagnostic cutoffs). For example, we observe increased mortality in patients with INR 1.2-1.49, but similar patterns emerge across multiple parameters, suggesting meaningful pathophysiological changes in continuous variables despite not meeting arbitrary diagnostic thresholds.
- **Progressive pre-sepsis**: A clinically significant proportion of detected patients who develop alterations compatible with sepsis 24–48 hours after initial detection. This trajectory validates the predictive window and confirms that pre-sepsis represents a dynamic state with genuine risk of progression to organ dysfunction.

These trajectories reveal two distinct phenomena within “pre-sepsis”: true “at-risk-of-sepsis” patients (infected non-septic and aborted sepsis groups) whom we can identify and prevent from progressing, versus patients with genuine early organ dysfunction that current binary cutoffs fail to capture (sub-threshold and progressive pre-sepsis groups). The latter raise Gerard et al.‘s provocative question: is “pre-sepsis” actually the real initial diagnosis of sepsis? [1].

This distinction has profound clinical implications. While optimal infection management should theoretically apply to all patients, clinical reality shows that sepsis diagnosis triggers systematically intensive care—enhanced monitoring, goal-directed resuscitation, early vasopressor support, and proactive non-invasive respiratory strategies—that is not routinely applied to non-septic infections. Critically, our pre-septic patients—despite demonstrating risk or subclinical dysfunction—would not meet criteria for sepsis bundles or intensive protocols under current guidelines. The 24-hour prediction window enables prospective intensified monitoring allowing more timely interventions during the pre-sepsis phase—exactly what Gerard et al. identify as necessary to shift from observing consequences to targeting causes [1]. The existence of this substantial pre-sepsis population (representing the majority of our detections) suggests that this is not merely a theoretical construct but a common, clinically significant state overlooked by current definitions.

The challenge ahead requires reconceptualizing sepsis as a continuum rather than a threshold phenomenon. The implications are profound: (1) what we call “pre-sepsis” may include both preventable risk states and unrecognized early sepsis; (2) we need continuous rather than binary diagnostic approaches; (3) interventions must be tailored to distinct trajectories rather than applied uniformly. Can we identify pre-sepsis endotypes analogous to those described for established sepsis? Should intervention strategies differ between true “at-risk” states versus subclinical organ dysfunction? Gerard et al. correctly note that current Sepsis-3 definitions, anchored in organ dysfunction, may represent intervention “too late” [1,4]. The challenge now is translating the pre-sepsis concept into actionable clinical practice: developing consensus criteria, identifying real-time biomarkers (such as the CO pathway proposed by the authors), and designing trials testing trajectory-specific interventions in this critical window.

Gerard et al.‘s framework provides essential theoretical grounding for this paradigm shift from threshold-based to trajectory-based sepsis diagnosis and management—a shift our clinical data supports as both necessary and feasible.
